# Prophylactic HIPEC with radical D2 gastrectomy improves survival and peritoneal recurrence rates for locally advanced gastric cancer: personal experience from a randomized case control study

**DOI:** 10.1186/s12885-019-6125-z

**Published:** 2019-09-18

**Authors:** Maneesh Kumarsing Beeharry, Zheng-Lun Zhu, Wen-Tao Liu, Xue-Xin Yao, Min Yan, Zheng-Gang Zhu

**Affiliations:** Department of Surgery, Ruijin Hospital affiliated Shanghai Jiao Tong University School of Medicine, Shanghai, 200025 China

**Keywords:** Advanced gastric Cancer, HIPEC, Cisplatin, Procedure morbidity, DFS

## Abstract

**Background:**

To investigate the implications of prophylactic intraoperative Hyperthermic Intraperitoneal Chemotherapy (HIPEC) with D2 radical gastrectomy for locally advanced Gastric Cancer (AGC) in a randomized case control study.

**Method:**

Eighty consecutive patients with locally AGC were randomly separated into 2 groups: HIPEC group (Curative Resection + intraoperative HIPEC with cisplatin 50 mg/m^2^ at 42.0 ± 1.0 °C for 60 min) and Control group (Curative Resection only). Intraoperative and post-operative events, clinical recovery, morbidity and the disease-free survival (DFS) rates were closely monitored.

**Results:**

Faster recovery of bowel function (43 ± 5 h vs 68 ± 7, *P* < 0.05) and shorter postoperative stay (8d vs 14d, *P* < 0.05) were noted in the HIPEC group. Among the 40 HIPEC group patients, the highest intracranial temperature recorded during the procedure was 38.2 °C but the patient made an eventless recovery. Mild renal dysfunction, hyperbilirubinemia and mild liver dysfunction were recorded in the HIPEC group but their incidences were found to be statistically insignificant when compared with the control group (*P* > 0.05). The 3 year DFS rate analysis showed that the prophylactic HIPEC group had a higher DFS rate (93% vs 65%, *P* = 0.0054). The peritoneal recurrence rate was lower in the HIPEC group (3% vs 23%, *P* < 0.05).

**Conclusion:**

Prophylactic HIPEC with radical D2 Gastrectomy improves survival and peritoneal recurrence rates for AGC with favorable post-operative recovery at low and acceptable morbidity.

## Background

Gastric cancer (GC), one of the main leading causes of cancer-related mortality worldwide [1], is associated with a relatively high risk of peritoneal carcinomatosis (PC) with a prevalence rate of 5–20%, as a result of which around 50% of patients with potentially curable advanced gastric cancer (AGC) die from cancer recurrence in the peritoneum [2]. Nevertheless, 15 to 50% of AGC patients with serosal involvement present peritoneal dissemination at surgical exploration [3] and such cases have been associated with poor survival as shown by many phase III trials that reported median survival ranging from 1 to 13.8 months [3–8] and no survivor at 5 years [9, 10].

In a study performed on 1108 GC patients undergoing radical D2 gastrectomy, almost 50% of the patients had tumor recurrence, with 15.5% manifesting metachronous PC after a median time of 17.7 months post-surgery and upon further investigation, the degree of serosal involvement, extent of nodal metastasis and tumor pathological subtype (signet cell or undifferentiated carcinoma) were found to be risk factors leading to disease progression to PC [11, 12]. In the Japanese General Rules of Gastric Cancer Treatment, PC is separated into two categories: P0/Cy1 and P1 [13]; P0/Cy1 indicating positive peritoneal wash cytology without macroscopic PC while P1 indicates the macroscopic PC with or without positive peritoneal cytology and it has been shown that the prognosis of P0/Cy1 GC is similar to P1 GC [14]. Because of the limited efficacy of systemic chemotherapy to control PC, other treatment strategies including regional approaches have been explored and hence, Hyperthermic intra-peritoneal chemotherapy (HIPEC) has shown to effectively control PC from ovarian or mucinous appendiceal cancer [15–17]. A meta-analysis based on the evaluation of 280 studies analyzing the implications of HIPEC for GC patients with serosal invasion indicated that HIPEC could potentially allow a better prognosis in patients at acceptable rate of complications [18].

Our personal experience shows that HIPEC shows promise in the management of AGC as prophylaxis to peritoneal recurrence [19]. However, despite the supposed high efficacy of intraoperative HIPEC combined with radical resection, this multimodal approach has yet not been considered as a routine practice for AGC clinical management and this might be a result of the high procedure related morbidity. The early attempt from Fujimura et al. reported a morbidity of 50% and a reoperation rate of 33.3% [20]. Nevertheless, later studies were more optimistic with morbidity rates ranging from 9.6 to 55.6%, and mortality rates of 0 to 14.3% [21]. In a meta-analysis of randomized and high quality non-randomized studies investigating the role of HIPEC in the GC [22], Desiderio et al investigated a total of 2520 cases and for the GC patients without PC, the overall survival rates between the HIPEC and control groups at 3 or 5 years resulted in favor of the HIPEC group (RR = 0.82, *P* = 0.01). However, HIPEC was associated with significantly higher risk of complications for both patients with PC (RR = 2.15, *P* < 0.01) and without (RR = 2.17, *P* < 0.01) [23] . This increased risk in the HIPEC group was related to systemic drugs toxicity while anastomotic leakage rates were found to be similar between groups.

Hence, in this randomized case-control study, we have investigated the feasibility and morbidity of intraoperative HIPEC as prophylaxis against PC in AGC patients and compared the survival rates in a small cohort.

## Methods

### Patients selection

Between December 2014 and June 2015, consecutive AGC patients were carefully screened and 80 GC patients conforming with the following criteria were included for the study: (1) aged between 18 and 76 years old; (2) Primary GC; (3) Preoperative staging (Computed Tomography or endoscopic ultrasonography) revealing lesion(s) infiltrating the sub-serosal layers and above (T staging ≥ T3); (4) Karnofsky performance status (KPS) > 50; (5) Peripheral blood white blood cells (WBC) count ≥3500/mm^3^, platelet count (PLT) ≥80,000/mm^3^ and hemoglobin count (Hb) ≥ 90 g/L; (4) Normal liver function, with bilirubin ≤2 times the upper limit of normal (ULN), and aspartate aminotransferase (AST) and alanine aminotransferase (ALT) ≤2 × ULN; (5) Normal renal function, with serum creatinine (SCr) ≤ 1.5 mg/dl; (6) Full cardiac and pulmonary assessment without obvious surgery contra-indications. Patients with positive cytology during the laparoscopic examination were excluded from the study.

Patients conforming to the requirements signed informed consent. The study was approved by the institutional review board and the ethics committee of Shanghai Jiao Tong University School of Medicine affiliated Shanghai Ruijin Hospital.

### Randomization

Pre-printed group allocation chits were sealed in 80 envelopes which would randomly separate 80 consecutive patients into 2 groups: the HIPEC group (*n* = 40) and the Control group (*n* = 40) whereby the HIPEC group would include patients who would undergo Radical Surgery (RS) followed by intraoperative HIPEC while the Control group would only undergo RS. Randomization was performed on the day of surgery just before the operation would start.

All RS + HIPEC procedures were performed by a designated team of surgeons. After anesthesia, laparoscopic exploration with primary peritoneal lavage cytology was conducted to rule out occult peritoneal dissemination. All of the subjects underwent standardized radical gastrectomy with D2 lymphadenectomy. After anastomosis, the panel of trained and experienced surgeons with a designated anesthesiologist would conduct the intraoperative HIPEC using the RanD Performer® HT perfusion device (RanD Co. Ltd., Italy). The open coliseum technique HIPEC was adopted for optimal thermal homogeneity and spatial diffusion with 50 mg of cisplatin (CDDP) per liter of saline perfusate, maintained at 42.0 ± 1.0 °C. The perfusion rate was controlled between 600 and 1000 mL/min and total perfusion time was 60mins.

During the procedure, 6 temperature probes placed at the inlet, outlet, upper right, upper left, lower right and lower left abdomen were used to dynamically monitor temperature during the procedure. 2 other probes were used by the anesthesiologists to allow continuous monitoring of the patient’s arterial blood and intracranial temperature by using an intranasal probe. For statistical purposes, temperatures were tabulated at 10mins intervals during the procedure. In case of dangerous hyperthermia, including body, intraperitoneal and intracranial temperatures exceeding 44 °C, bleeding and unstable vitals, the whole process would be stopped.

### Parameters and post-operative follow-up

The basic clinico-physiological parameters such as temperature, blood pressure, respiration rate and heart rate were monitored on a daily basis with blood routine, liver and renal functions and electrolytes examinations repeated at days 1, 3 and 7. Other clinical parameters involved the time of nasogastric extubation, number of hours to recovery of bowel function (flatus or bowel movement), pain management, incidence of adverse effects (AE) or severe adverse effects (SAE) such as peritonitis, myelosuppression, anastomotic leak, bowel obstruction, intestinal necrosis, renal or hepatic dysfunctions, jaundice, loss of hair, abdominal distress, surgical wound dehiscence and the duration of postoperative stay of subject. The data recording was performed by a group of nurses blinded to the randomization result.

### Post-operative follow-up and end-points

The primary endpoint was 3 years post-surgery. In-hospital perioperative complications were studied as secondary endpoints. Postoperative complications were classified based on the therapy-oriented severity grading system (TOSGS) and NCI Common Terminology Criteria (CTC) for Adverse Events version 4.0. Postoperative morbidity was analyzed according to the Dindo-Clavien classification for surgical complications [24]. The survival study involved comparison of the disease-free survival (DFS) in both groups. The patients from both groups were administered to 6 regimens of standard dosage of the XELOX regimen starting within 1 month after surgery (Regimen: Oxaliplacin 130 mg/m^2^ ivgtt d1 + Xeloda 1500 mg/m^2^ bid po d1–15, Q3W).

### Statistical analysis

All data were systematically collected to establish a comprehensive database of clinical records, surgical and pathology reports, image examination and laboratory reports, and follow-up records. The data were analyzed by SPSS software for windows, version 19.0 (SPSS Inc., Chicago, IL, USA). The Kaplan–Meier survival curve was plotted for survival analysis and the log rank test was utilized to identify difference between curves. A two-sided *P* < 0.05 was considered as statistically significant.

## Results

**Fig. 1:**
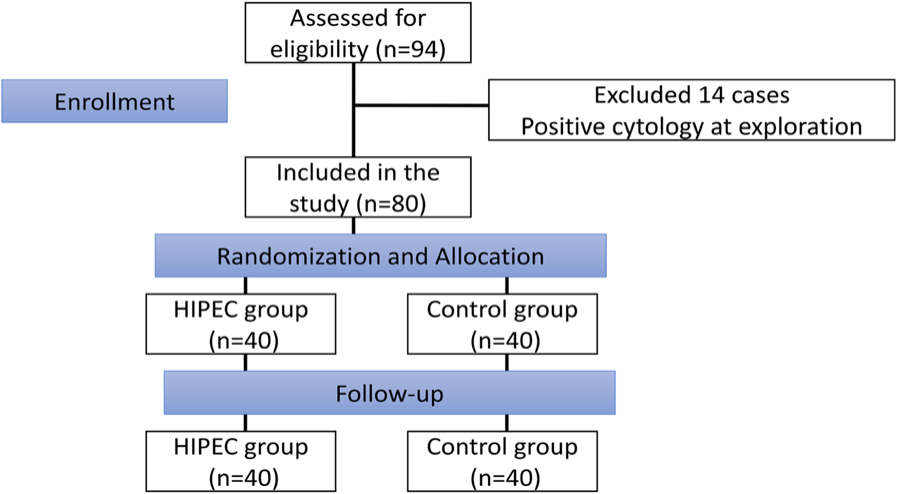
Consort Diagram for the experiment

Clinico-pathologic Characteristics of the subjects Between December 2014 and June 2015, 94 consecutive patients conforming to the requirements of the study were initially included in the study. However, 14 patients were excluded from the study due to positive cytology after the laparoscopic exploration (see Fig. 1 for the study consort diagram).

There were 46 male and 34 female patients with a mean age of 59 years for the HIPEC group and 58 years for the control group (*P* > 0.05). The median KPS was over 80% for all patients (Table 1). All the physiological and biochemical blood parameters to be investigated in the patients at the various time-points were weighed and recorded at baseline and no differences were found, suggesting that the model was well-balanced.

**Table 1:** Clinicopathological characteristics of the subjects

Characteristics	HIPEC Group (*n* = 40)	Control group (*n* = 40)	*P* value
Sex (n, %)			
Male	23 (57.5)	23 (57.5)	1
Female	17 (42.5)	17 (42.5)	
Mean age (years, range)	59 ± 10 (23–72)	58 ± 10 (36–74)	0.95
Mean KPS score (range)	85 ± 6 (70–90)	85 ± 6 (70–90)	0.979
Histopathology (n, %)			
Adenocarcinoma (poor or moderately differentiated) (n, %)	25 (62.5)	26 (65)	1
Adenocarcinoma (Mucinous, signet cell or other type) (n, %)	15 {37.5)	14 (35)	
Pre-operative Blood physiological and biochemical Parameters			
WBC (10^9^/L)	5.7 ± 1.4	5.7 ± 1.4	0.785
RBC (10^12^/L)	4.2 ± 0.6	4.3 ± 0.5	0.78
Hb (g/L)	130 ± 14	127 ± 20	0.339
Alb (g/L)	40 ± 5	39 ± 4	0.152
AST (IU/L)	20.4 ± 6.3	19.4 ± 8.1	0.094
ALT (IU/L)	20.3 ± 9.3	17.1 ± 8.2	0.854
TB (umol/L)	13.2 ± 4.6	12.9 ± 3.9	0.456
DB (umol/L)	2.2 ± 1.0	2.3 ± 0.08	0.626
SCr (umol/L)	73.5 ± 15.2	76.8 ± 27.3	0.134
K^+^ (mmol/L)	3.95 ± 0.45	3.84 ± 0.39	0.456
Na^+^ (mmol/L)	141 ± 5	140 ± 4	0.334

*KPS* Karnofsky Performance Score, *WBC* White Blood Cell Count, *RBC* Red Blood Cell Count, *Hb* Hemoglobin, *Alb* Albumin, *AST* Aspartate Aminotransferase, *ALT* Alanine transaminase, *TB* Total Bilirubin, *DB* Direct Bilirubin, *SCr* Serum Creatinine, *K*^*+*^ Potassium Ion, *Na*^*+*^ Sodium Ion, *cT3* clinical evidence of sub-serosal invasion, *cT4* clinical evidence of serosal invasion

There was no significant difference in the histology of the lesions, the surgical procedure (distal or total gastrectomy), rate of lymph node metastasis or post-operative TNM staging of the subjects (Table 2).

**Table 2:** Surgical procedures and pathological characteristics of the subjects

Characteristics	HIPEC Group (*n* = 40)	Control group (*n* = 40)	*P* value
Surgical Procedures			
Total Gastrectomy	13 (32.5)	17 (42.5)	0.489
Distal Gastrectomy	27 (67.5)	23 (57.5)	
Postoperative T Staging: pT3 versus pT4a			
pT3	2	2	1
pT4a	38	38	
Postoperative pN Staging			
N0	3	9	0.362
N1	7	8	
N2	10	9	
N3a	10	8	
N3b	10	6	
Rate of Lymph Node Metastasis (%)	27.8 ± 23.3	33.1 ± 28.1	0.06
Postoperative pTNM Staging			
IIA	0	1	0.242
IIB	2	8	
IIIA	8	7	
IIIB	11	10	
IIIC	19	14	

### Safety implications of HIPEC

#### Monitoring of the different temperature probes during HIPEC revealed no significant fluctuations in the intracranial and peripheral blood temperatures

During the 60 mins HIPEC circulation time, the blood (T_blood_) and intracranial (T_intracranial_) temperatures were also recorded at 10 min intervals and the results are shown in Table 3. There was no significant statistical difference between the blood and intracranial temperatures at all time-intervals when the intraperitoneal temperature was maintained at 42.0 ± 1.0 °*C. maximum* blood and intracranial temperature was 38.1 °C both recorded at 50 mins in 1 patient but the latter made an eventless recovery from surgery.

**Table 3:** Comparison of blood and intracranial temperatures during the HIPEC procedure

	Start of HIPEC T_0_	During HIPEC					
		10 mins	20 mins	30 mins	40 mins	50 mins	60 mins
T_blood_(°C)	36.7 ± 0.3	36.5 ± 0.8	36.7 ± 1.0	36.5 ± 0.1	36.5 ± 0.1	36.6 ± 0.1	36.6 ± 0.2
T_intracranial_(°C)	36.6 ± 0.2	36.4 ± 0.3	36.8 ± 0.2	36.5 ± 0.2	36.5 ± 0.1	36.7 ± 0.3	36.6 ± 0.2
*P* value	0.638	0.253	0.666	0.576	0.123	0.064	0.322

#### The 7 days post-operative temperature monitoring showed that there was no fever due to the intraoperative hyperthermia

As shown in Table 4, there was a significant difference in the temperatures recorded on days 1 and 2 post-op: the temperature of the control group was significantly higher than the HIPEC group (37.7 versus 36.8 °C on Day 1 and 37.2 versus 36.7 °C on Day 2). No post-operative fever was recorded in the HIPEC group while in the control group, temperatures higher than 38.0 °C were recorded in 2 patients on day 1 and 15 patients on day 2. However, there was no high temperature recorded from days 3 to 7 (*P* > 0.05). The patients presenting with temperatures higher than 38.0 °C had slightly elevated white blood counts but without any complaint of any related discomfort.

**Table 4:** Post-operative maximum daily temperatures of all the 80 subjects

	Post-operative Maximum Body Temperature (°C)						
	Day 1	Day 2	Day 3	Day 4	Day 5	Day 6	Day 7
HIPEC Group (*n* = 40)	36.8 ± 0.4	36.7 ± 0.3	36.8 ± 1.6	36.8 ± 0.2	36.6 ± 0.2	36.7 ± 1.3	36.8 ± 0.2
Control Group (*n* = 40)	37.7 ± 0.6	37.2 ± 0.2	36.9 ± 0.2	36.9 ± 0.2	36.8 ± 0.3	36.7 ± 0.2	36.7 ± 0.2
*P* value	0.001	0	0.404	0.638	0.572	0.129	0.176

The patient who had blood and intracranial temperatures exceeding 38 °C during the HIPEC procedure had a maximum temperature of 37.4 °C on day 1 and a mean of 36.94 °C over the post-operative week.

Overall, the hyperthermic activities during the HIPEC procedure did not cause hyperthermia or hypothermia in the patients. No patient from the HIPEC group manifested post-operative fever or any complication that could be related to the intraoperative hyperthermia.

#### Post-operative morbidity and mortality

With all post-operative morbidity assessed according to CTCAE v4.0, of the 40 patients, adverse-effects (AE) were recorded in 14 patients, 3 in the HIPEC group (7.5%) and 11 in the control group (15%). There was no 30-day mortality in both groups. Mild neutropenia was recorded on post-op day 1 in 3 patients, 1 in the HIPEC group and 2 in the Control group. However, without specific therapy, the blood panels of the patients were normal on day 3 and stayed so on days 7 and 30. Mild bowel obstruction or intestinal ileus was noted in 1 patient from the Control group but the situation resolved after 2 days of conservative therapy. Mild renal toxicity was recorded in 2 patients, one from each group but renal function had resolved by day 7 without prompting for any further specific intervention. Mild abnormalities in liver function were recorded in 2 patients from the Control group which eventually resolved after courses of immediate hepatoprotective treatment. Mild hyperbilirubinemia was noted in 1 HIPEC case and 2 control cases and the patients were treated conservatively and the total bilirubin count was back to normal in both cases at day 7. 1 case from the control group manifested with surgical wound dehiscence with localized infection. Nevertheless, there was no incidence of post-operative bleeding, gastroparesis, HIPEC-related peritonitis, anastomotic leak, intestinal necrosis, diarrhea or allergic reactions in both groups (Table 5).

**Table 5:** Comparison of the post-operative blood physiological and biochemical parameters of the HIPEC and control groups

Parameters	Groups	Day 1	Day 3	Day 7	Day 30
WBC (10^9^/L)	HIPEC (*n* = 40)	14.01 ± 3.45	7.11 ± 2.51	6.08 ± 1.61	4.97 ± 1.23
	Control (*n* = 40)	13.34 ± 2.41	7.03 ± 2.13	5.88 ± 1.46	4.86 ± 1.39
	*P* Value	0.09	0.25	0.538	0.823
RBC (10^12^/L)	HIPEC (*n* = 40)	4.07 ± 0.54	3.87 ± 0.63	3.99 ± 0.55	4.05 ± 0.51
	Control (*n* = 40)	4.24 ± 0.47	4.01 ± 0.56	3.94 ± 0.49	4.03 ± 0.52
	*P* Value	0.943	0.08	0.252	0.854
Hb (g/L)	HIPEC (*n* = 40)	121.60 ± 7.37	118.05 ± 19.59	125.08 ± 12.11	121.50 ± 22.12
	Control (*n* = 40)	123.53 ± 17.54	116.88 ± 19.69	114.95 ± 16.82	128.30 ± 12.43
	*P* Value	0.899	0.1	0.518	0.335
Alb (g/L)	HIPEC (*n* = 40)	30.93 ± 3.33	37.68 ± 4.07	38.65 ± 4.86	18.80 ± 8.33
	Control (*n* = 40)	31.28 ± 3.43	36.33 ± 6.25	39.92 ± 7.38	19.25 ± 8.00
	*P* Value	0.6	0.86	0.323	0.640
AST (IU/L)	HIPEC (*n* = 40)	21.33 ± 10.49	28.95 ± 9.92	20.15 ± 11.1	21.28 ± 3.06
	Control (*n* = 40)	22.98 ± 9.25	27.42 ± 11.45	21.2 ± 16.07	21.62 ± 2.78
	*P* Value	0.09	0.111	0.347	0.759
ALT (IU/L)	HIPEC (*n* = 40)	34.85 ± 19.8	26.38 ± 16.15	21.03 ± 10.49	21.30 ± 6.47
	Control (*n* = 40)	29.53 ± 20.05	19.92 ± 15.10	22.98 ± 17.9	21.32 ± 7.34
	*P* Value	0.154	0.1	0.149	0.786
TB (umol/L)	HIPEC (*n* = 40)	16.24 ± 4.59	16.19 ± 5.42	15.75 ± 5.43	11.73 ± 4.13
	Control (*n* = 40)	12.91 ± 3.85	17.07 ± 0.88	17.01 ± 8.03	12.64 ± 2.83
	*P* Value	0.456	0.242	0.121	0.71
DB (umol/L)	HIPEC (*n* = 40)	3.72 ± 1.73	3.11 ± 1.69	3.48 ± 1.80	2.27 ± 1.18
	Control (*n* = 40)	3.64 ± 1.51	3.45 ± 0.91	3.12 ± 2.12	2.75 ± 1.02
	*P* Value	0.511	0.84	0.902	0.931
SCr (umol/L)	HIPEC (*n* = 40)	70.62 ± 27.1	68.13 ± 14.6	71.05 ± 17.93	68.42 ± 16.42
	Control (*n* = 40)	72.37 ± 25.4	63.31 ± 18.1	64.54 ± 13.69	67.27 ± 12.9
	*P* Value	0.319	0.569	0.199	0.09
K+ (mmol/L)	HIPEC (*n* = 40)	3.66 ± 0.49	3.62 ± 0.52	3.85 ± 0.23	3.64 ± 0.19
	Control (*n* = 40)	3.69 ± 0.39	3.92 ± 0.51	3.81 ± 0.39	3.78 ± 0.21
	*P* Value	0.89	0.763	0.743	0.452
Na + (mmol/L)	HIPEC (*n* = 40)	137.23 ± 2.89	139.28 ± 2.97	138.57 ± 2.54	139.12 ± 1.89
	Control (*n* = 40)	137.25 ± 2.62	141.25 ± 1.63	138.25 ± 2.34	138.82 ± 1.97
	*P* Value	0.924	0.897	0.824	0.813

*WBC* White Blood Cell Count, *RBC* Red Blood Cell Count, *Hb* Hemoglobin, *Alb* Albumin, *AST* Aspartate Aminotransferase, *ALT* Alanine transaminase, *TB* Total Bilirubin, *DB* Direct Bilirubin, *SCr* Serum Creatinine, *K+* Potassium Ion, *Na+* Sodium Ion

When the cases with complications were independently compared with the corresponding incidence in the Control group using Pearson Chi Square test (Table 6), it was found that there was no statistically significant difference between the 2 groups, implying that the post-operative complications recorded in the HIPEC group was not related to the HIPEC procedure itself (*P* = 0.556).

**Table 6:** Most common grade 3–5 adverse events and adverse events of special interest with cisplatin (related and unrelated events; safety population); Results are presented as number of patients and all the side-effects were evaluated according to CTCAE v4.0

Complication	HIPEC Group (*n* = 40)	Control Group (*n* = 40)
Neutropenia	1	2
Anastomotic Leak	0	1
Bowel Obstruction	0	1
Renal Toxicity	1	1
Liver Function Dysfunction	0	2
Hyperbilirubinemia	1	2
Post-operative Infection	0	1
Surgical Wound Dehiscence	0	1
Total Perioperative Side-effects	3	11

### Post-operative clinical management parameters

The median time to nasogastric tube removal was 1 day in both groups (*P* > 0.05). Interestingly, the time to recovery of bowel function was shorter in the HIPEC group (42.9 versus 67.8 h, *P* < 0.05), and subsequently the time to liquid diet was shorter in the HIPEC group (3.03 versus 4.02 days, *P* < 0.05). The time to surgical sutures removal was 7 days in both groups (*P* > 0.05). Eventually, the overall duration of stay after the procedure was shorter in the HIPEC group (8.15 versus 14.08 days, *P* < 0.05).

### Survival analysis

**Fig. 2:**
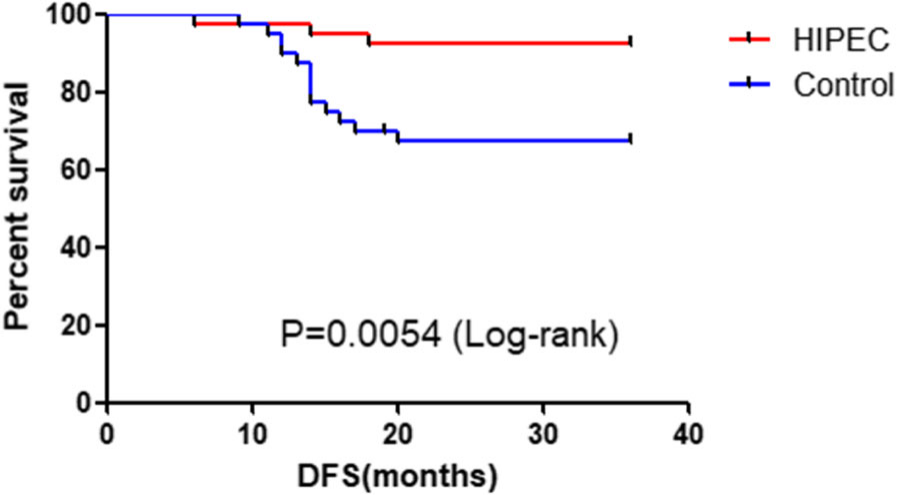
Short-term Disease free survival (DFS) analysis of the HIPEC versus Control Group

During a median follow-up of 32 months, 4/80 died of disease progression (1 patient from each group died of brain metastasis undetected at presentation and 1 patient from each group died of extensive metastasis). At 3 years of follow up, from the HIPEC group, there were 3/40 cases of disease progression (1 case of brain metastasis at 6 months, 1 case of extensive with peritoneal metastasis at 14 months and 1 case of retroperitoneal lymph node metastasis at 18 months); in the Control group, there were 14/40cases of disease progression (4 cases presented with liver metastasis with possible peritoneal dissemination at 19, 14, 12 and 13 months; 5 cases of peritoneal metastasis at 9, 12, 14, 15, 17 months;1 case of brain metastasis at 11 months; 1 case of extensive metastasis at 14 months; 3 cases with retroperitoneal lymph node metastasis at 14, 16, 20 months) and the difference in the disease free survival (DFS) was statistically significant (93% vs 65%, *P* = 0.0054) (see Fig. 2). The peritoneal recurrence rate of the control group was much higher than the HIPEC group (3% vs 23%, *P* < 0.05).

## Discussion

The mortality rate after intraoperative HIPEC has been reported to be ranging from 0.9 to 5.8% [24]. Recent studies showed that perioperative hypothermia manifests in more than 70% of patients and eventually can lead to several complications such as augmented intraoperative blood loss due to impaired platelet function and clotting factor enzyme function, peripheral vasoconstriction increasing the incidence of surgical wound infection due to reduction of subcutaneous oxygen tension and an impairment in immune function, or increase in heart rate and oxygen demand in the presence of shivering [25–29]. Therefore, thermoregulation plays a very important role during the HIPEC procedure. Nevertheless, the risks associated with hyperthermia have also been linked with direct complications on the gastrointestinal and cardiovascular functions. Significant fluctuations in the temperature during the procedure could lead to dangerous outcomes such as intestinal necrosis, hemorrhage, renal and hepatic dysfunctions and in case of intracranial hyperthermia, there could be cognitive impairments which could lead to electrophysiological alterations. In this study, we monitored the changes in the intraperitoneal, blood and intracranial temperatures of the HIPEC patients when the target therapeutic intraperitoneal temperature was set to 42.0 °C and the results showed no significant difference found in the three correlated sets temperatures, suggesting that the continuous flow during the HIPEC procedure not only allowed stable hyperthermia in the peritoneal cavity but simultaneously decreased the risks of hyperthermia in the peripheral blood and intracranial environment. In contrast, while monitoring the daily temperatures of the 80 subjects within one week after the procedure, no significant different was recorded in the overall trend between the 2 sets of patients, suggesting that the application of HIPEC did not contribute to post-operative fever or related morbidity.

HIPEC has also been associated with postoperative fever, severe local and/or systemic infection, intestinal or anastomotic leakage, intestinal obstruction, hemorrhage, HIPEC-related peritonitis, myelosuppression etc. Literature reports about 40% of patients developing at least one postoperative in hospital complication [30] with a low incidence of need for organ support and other significant critical care interventions [31]. Previous research have recognized some rather common procedure-related complications such as anastomotic leaks (0–9%), intra-abdominal abscesses (0–37%), intestinal perforation (0–10%), fistulas (0–23%), and prolonged ileus (0–86%) [15–17]. Nevertheless, other morbidity such as post-operative bleeding, bile leaks, pancreatitis, wound infections, acalculous cholecystitis, mesenteric ischemia, mechanical intestinal obstruction have also been reported, but with lower incidence rate [16–20]. However, in our study, there was no incidence of these complications and when compared to the control group, the post-operative morbidity rate was not found to be statistically different, further affirming that HIPEC is not a significant risk factor associated with postoperative complications. We did not record any HIPEC related mortality in our study.

The HIPEC patients showed relatively faster gastrointestinal function recovery with flatus or passing of stool at around 42.9 h post-operatively (compared to 67.8 h in the control group). They were hence started on liquid diets earlier than the control group. The HIPEC patients did not complain of any episodes of intestinal cramps or abdominal discomfort after the surgery while the control group patients complained about intermittent intestinal cramps that subsided after passing gas. The time to suture removal was also 7 to 8 days in both groups, suggesting that the hyperthermia during the surgery had not compromised with the healing process of both the incision and anastomosis. Moreover, the overall duration of stay after the procedure was shorter in the HIPEC group (8.2 versus 14.1 days, *P* < 0.05). These results suggest that the patients undergoing HIPEC had quicker postoperative recovery with comparably lower incidence of postoperative complications.

The perioperative clinical management of AGC patients with high risk of intraperitoneal dissemination requires the prophylactic curative effect of HIPEC. In this study, we have assessed the various aspects of the safety issues that were initially linked with the routine practice of HIPEC and we have concluded that based on a randomized controlled approach, the combination of CS and HIPEC was efficient, safe and less risky as proposed in previous literature. Currently, there are few meta-analyses overviewing the outcomes of previous trials using prophylactic HIPEC [32–36] among which 2 meta-analyses included only patients receiving HIPEC in the experimental arm [18, 37] and both of them did not show any significant increase in the rate of post-operative morbidity.

In this study, we have conducted a short term DFS investigation and out results concluded that from the HIPEC group, there were 4/40 cases of disease progression (1 case of brain metastasis at 6 months, 1 case of extensive metastasis at 12 months, 1 case of liver metastasis at 14 months and 1 case of retroperitoneal lymph node metastasis at 18 months); in the non-HIPEC group, there were 14/40 cases of disease progression (4 cases presented with liver metastasis with possible peritoneal dissemination at 19, 14, 12 and 13 months; 5 cases of peritoneal metastasis at 9, 12, 14, 15, 17 months;1 case of brain metastasis at 11 months; 1 case of extensive metastasis at 14 months; 3 cases with retroperitoneal lymph node metastasis at 14, 16, 20 months) and the difference in the disease free survival (DFS) was statistically significant (*P* = 0.0016). The peritoneal recurrence rate of the control group was higher than the HIPEC group (*P* < 0.05)., suggesting that Intraoperative HIPEC can effectively improve the survival outcome of advanced GC patients at risk of peritoneal dissemination and decrease the rate of peritoneal recurrence.

In an overview of 10 RCTs, Sun et al. [18] suggested a significant advantage in survival with the use of HIPEC. In contrast, while investigating 16 RCTs, Mi et al. [37] reported a significant improvement in the 1, 2, 3, 5 and 9-year survival and a decrease in the peritoneal recurrence rates at 2, 3 and 5 years in patients who received HIPEC.

There were 2 mortalities recorded in our study, both patients developing brain metastasis during the follow-up period. Of the 40 patients from the control group, 6 patients had disease progression with 4 patients presenting with liver metastasis with possible peritoneal metastasis and the other 2 with retroperitoneal lymphadenopathy with peritoneal metastasis. The HIPEC group patients are still stable. The median time to disease progression was around 13 months, which is consistent with previous literature. There have been speculations about the high concentration of hyperthermic drugs in the abdominal cavity facilitated the absorption by the peritoneum and the circulatory system through the portal vein and retroperitoneal lymphatic system, which is very consistent with route of GC metastasis, therefore controlling micro-metastases in the lymphatic system and liver [38]. However, more concrete evidence is required to sustain this hypothesis.

### Limitations of the study

This study has been presented in the form of an observational study due to its limitations as a fully structured randomized controlled trial. In this study, we have tried to investigate the feasibility and efficacy of HIPEC on consecutive 40 patients by comparing their outcomes with consecutive simultaneous 40 control subjects, all undergoing D2 radical gastrectomy. Our future prospects would be to follow-up the investigation with a larger and more structured prospective RCT approach.

## Conclusion

The rising incidence of GC around the world prompts for comprehensive multimodality treatment plans in order to improve AGC prognosis. The combination of intraoperative HIPEC with CS offers better clinical management of patients with high risk of secondary peritoneal carcinomatosis, favoring their post-operative recovery at low incidence of complications and eventually improving their survival rates.

## Data Availability

Data and material are available upon request.

## Abbreviations

AE: Adverse Effects
AGC: Advanced Gastric Cancer
ALT: Alanine transaminase
AST: Aspartate Aminotransferase
CDDP: Cisplatin
CTC: NCI Common Terminology Criteria
DB: Direct Bilirubin
DFS: Disease Free Survival
GC: Gastric Cancer
Hb: Hemoglobin; Alb Albumin
HIPEC: Hyperthermic Intraperitoneal Chemotherapy
KPS: Karnofsky Performance Score
PC: Peritoneal Carcinomatosis
RBC: Red Blood Cell Count
RS: Radical Surgery
SAE: Severe Adverse Effects
SCr: Serum Creatinine
TB: Total Bilirubin
TOSGS: Therapy-oriented severity grading system
WBC: White Blood Cell Count
