# Correction to: Prophylactic HIPEC with radical D2 gastrectomy improves survival and peritoneal recurrence rates for locally advanced gastric cancer: personal experience from a randomized case control study

**DOI:** 10.1186/s12885-019-6411-9

**Published:** 2019-12-30

**Authors:** Maneesh Kumarsing Beeharry, Zheng-Lun Zhu, Wen-Tao Liu, Xue-Xin Yao, Min Yan, Zheng-Gang Zhu

**Affiliations:** Department of Surgery, Ruijin Hospital affiliated Shanghai Jiao Tong University School of Medicine, Shanghai, 200025 China

**Correction to: BMC Cancer**

**https://doi.org/10.1186/s12885-019-6125-z**

Following publication of the original article [1], the authors reported the following errors/updates.

1. In the abstract, the Results and Conclusion have been updated as follows:
  **Results:** Among the 40 HIPEC group patients, the highest intracranial temperature recorded during the procedure was 38.2 °C but the patient made an eventless recovery. Mild renal dysfunction, hyperbilirubinemia and mild liver dysfunction were recorded in the HIPEC group but their incidences were found to be statistically insignificant when compared with the control group (*P* > 0.05). The initial post-operative analysis revealed shorter post-operative stay for in the HIPEC group but further analysis revealed that it was related to the incidence of postoperative complication. During a median follow-up time of 41 months, there were 9/39 and 15/38 cases of disease progression in HIPEC and Control groups respectively, with a more favorable 3-year DFS (76.9% vs 60.5%) and a lower peritoneal recurrence rate (5% vs 30%) in the HIPEC group.
  **Conclusion:** Prophylactic HIPEC with radical D2 Gastrectomy is safe and shows favorable survival and peritoneal recurrence rates for AGC with acceptable morbidity. Nevertheless, more structured multi-centered RCT should be carried out for more substantial evidence.
2. It was brought to our notice that the flow-diagram for the study scheme was not complete, hence we have updated it.

**Fig. 1:**
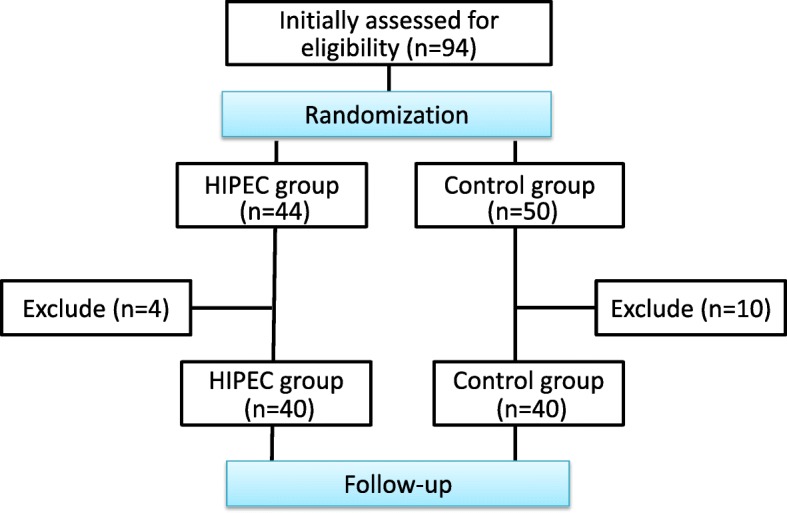
Study Overview

3. We have made some changes to the **Post-operative Clinical Management Parameters:** The median time to nasogastric tube removal was 1 day in both groups (*P* > 0.05). The time to recovery of bowel function was shorter in the HIPEC group (42.9 versus 67.8 h, *P* < 0.05), and subsequently the time to liquid diet was shorter in the HIPEC group (3.03 versus 4.02 days, *P* < 0.05). The time to surgical sutures removal was 7 days in both groups (*P* > 0.05). The post-operative duration of stay was shorter in the HIPEC group (9 versus 11 days, *P* < 0.05). However, upon further analysis, we discovered that the longer post-operative stay from the control group was due to the higher incidence of post-operative complication with 2 patients staying for around 28 and 80 days respectively.
4. We have updated the survival analysis data according to date of publication.
  Median follow-up for the entire cohort was 41 months (range 37–52 months). 3 patients were lost (1 from the HIPEC group at start, 1 from the control group at start and another at 26 months respectively) during the follow-up. From the HIPEC group, there were 9/39 cases of disease progression (1 case of brain metastasis at 6 months, 1 case of peritoneal metastasis at 14 months, 1 case of retroperitoneal nodal metastasis at 18 months, 1 case of extensive metastasis with liver metastasis at 16 months, 1 case of liver and pancreas metastasis at 31 months, 3 cases of recurrence at site of anastomosis at 15, 21 and 36 months and 1 death of unknown reason at 28 months). From the control group, there were 15/38 cases of disease progression (4 cases presented with liver metastasis with possible peritoneal dissemination at 13, 14, 19 and 31 months; 7 cases of peritoneal metastasis at 9, 14, 15, 17, 25, 29 and 30 months;1 case of brain metastasis at 11 months; 1 case of extensive metastasis at 14 months; 2 cases with bone and retroperitoneal lymph node metastasis at 13 and 20 months). Survival analysis revealed a 2 yr DFS of 86.7% vs 67.6% and a 3 yr DFS of 76.9% vs 60.5% for the HIPEC and Control groups respectively. The peritoneal recurrence rate of the control group was much higher than the HIPEC group (30% (11/37) vs 5% (2/38)).
5. With respect to the changes, we have updated Fig. 2.

**Fig. 2:**
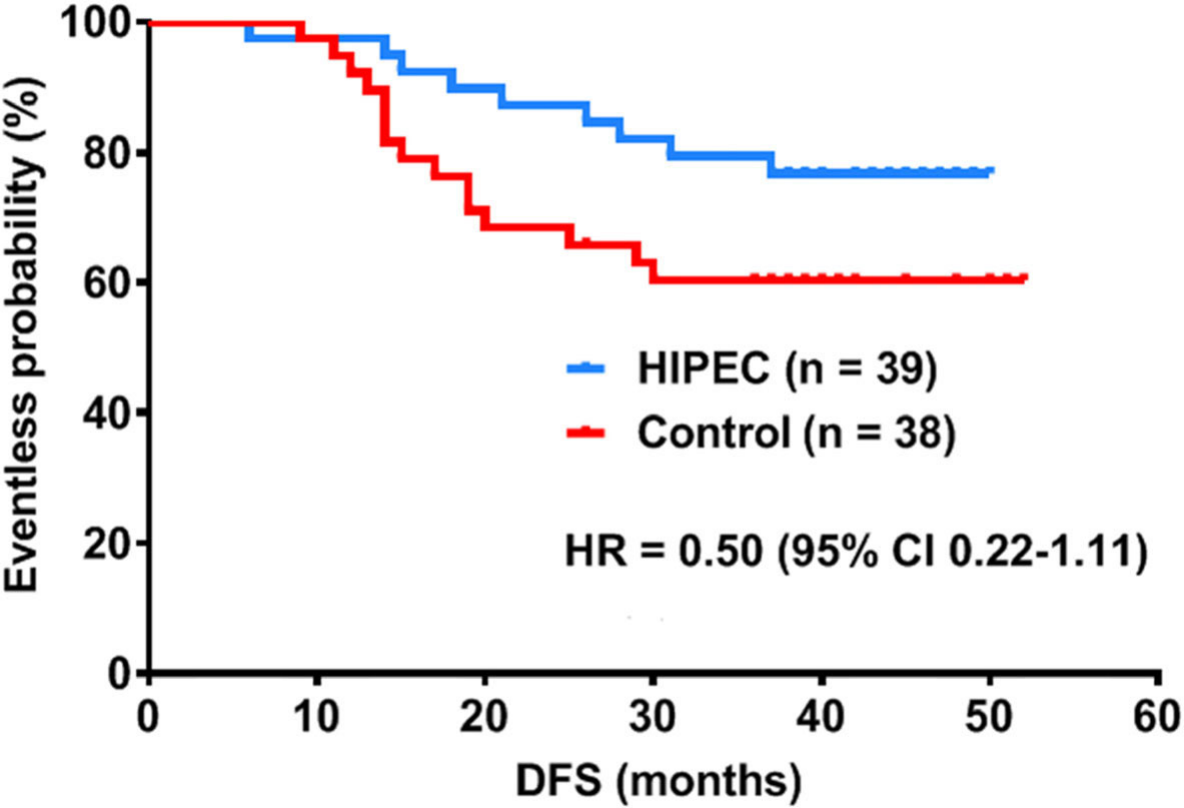
Short term survival analysis of the cohort

6. In the 3rd paragraph of the Discussion, we have made the following changes: During the analysis of the post-operative parameters, we discovered that the overall duration of post-operative stay was shorter in the HIPEC group (a median of 9 versus 11 days, *P* < 0.05). However, upon further analysis, we discovered that the longer post-operative stay from the control group was due to the higher incidence of post-operative complication with 2 patients staying for around 28 and 80 days respectively.
